# Differential Expression of Proteins Involved in Skin Barrier Maintenance and Vitamin D Metabolism in Atopic Dermatitis: A Cross-Sectional, Exploratory Study

**DOI:** 10.3390/ijms26010211

**Published:** 2024-12-30

**Authors:** Teresa Grieco, Giovanni Paolino, Elisa Moliterni, Camilla Chello, Alvise Sernicola, Colin Gerard Egan, Mariangela Morelli, Fabrizio Nannipieri, Santina Battaglia, Marina Accoto, Erika Tirotta, Silvia Trasciatti, Silvano Bonaretti, Giovanni Pellacani, Stefano Calvieri

**Affiliations:** 1 Dermatology Clinic, Department of Clinical Internal, Anesthesiological and Cardiovascular Sciences, Sapienza University of Rome, 00185 Rome, Italy; teresa.grieco@uniroma1.it (T.G.); elisa.moliterni@gmail.com (E.M.); c.chello@unicampus.it (C.C.); alvise.sernicola@uniroma1.it (A.S.); giovanni.pellacani@uniroma1.it (G.P.); stefano.calvieri@uniroma1.it (S.C.); 2 Unit of Dermatology and Cosmetology, IRCCS University Vita-Salute San Raffaele, 20132 Milan, Italy; 3 CE Medical Writing Srls, 56021 Pisa, Italy; colingegan@gmail.com; 4 Fondazione Pisana per la Scienza, 56017 Pisa, Italy; mmorelli190977@gmail.com; 5 Clinical Research, Abiogen Pharma SpA, 56121 Pisa, Italy; fabrizio.nannipieri@abiogen.it (F.N.); santina.battaglia@abiogen.it (S.B.); marina.accoto@abiogen.it (M.A.); erika.tirotta@abiogen.it (E.T.); 6 Galileo Research Srl, 56019 Pisa, Italy; silvia.trasciatti@galileoresearch.it (S.T.); silvano.bonaretti@galileoresearch.it (S.B.)

**Keywords:** atopic dermatitis, vitamin D receptor, skin barrier maintenance, protein expression

## Abstract

Atopic dermatitis (AD) is a chronic inflammatory skin disorder influenced by proteins involved in skin barrier maintenance and vitamin D metabolism. Using an intra-patient design, this study compared protein expression in intra-lesional (IL) and peri-lesional (PL) skin biopsies from AD patients and examined associations between protein levels, vitamin D status, and clinical features. Forty-four biopsies from twenty-two AD patients were analyzed using antibody microarrays targeting twelve proteins. IL samples had significantly higher total protein levels than PL samples, with a mean difference of 77.7% (*p* < 0.001). Several proteins, including cathelicidin, cingulin, occludin, filaggrin, and the vitamin D receptor, were upregulated in IL samples. Patients with vitamin D levels below 30 ng/mL showed higher expression of CYP24A (*p* = 0.054), alpha-catenin (*p* = 0.043), and haptoglobin (*p* = 0.033). Increased EASI scores (≥16) were associated with elevated expression of CYP24A (*p* = 0.024), CYP27B (*p* = 0.044), filaggrin (*p* = 0.027), occludin (*p* = 0.049), and claudin-1 (*p* = 0.052). Multivariate regression analysis identified significant correlations between protein expression, skin prick test positivity, and low vitamin D levels. These findings suggest that proteins related to epithelial barrier function and vitamin D metabolism are highly upregulated in IL skin regions, offering potential therapeutic targets for improving both skin barrier function and overall disease severity in AD patients.

## 1. Introduction

Atopic dermatitis (AD) is a chronic inflammatory skin condition that often coexists with other atopic disorders, including allergic rhinitis, food allergies, and asthma [1,2]. Affecting up to 20% of children and 14% of adults, the prevalence of AD varies across ethnicities and geographic regions [3]. The complex pathogenesis of this disease is primarily marked by alterations in the skin barrier, the immune system, and the microbiome. Epidermal barrier dysfunction plays an important role in the development of AD [4]. Filaggrin (FILA), a major epidermal protein, is essential for the structure and function of the stratum corneum (SC), the outermost layer of the skin, and is recognized as a key factor in the pathogenesis of AD [5]. The SC provides a physical barrier, serving as the first line of defense against environmental factors, pathogens, and allergens while also maintaining water homeostasis [6]. The role of FLG in keratinization (or cornification) is vital as the epidermis regenerates approximately every 28 days, completing the process of terminal differentiation [5]. Tight junctions (TJs) are another crucial component, consisting of proteins located between cells in the stratum granulosum of the skin that function to limit the passage of substances like water and larger molecules [7] and to prevent nerve fibers from extending to the skin’s surface, potentially helping to reduce itching [8]. TJs include transmembrane components such as claudins (CLD), occludins (OCLNs), and intracellular junctional adhesion molecules (e.g., zonula occludens proteins and cingulin, CING) [6]. 

Another key structure involved in maintaining strong cell–cell connections and facilitating effective communication is adherens junctions, which are formed by transcellular elements like E-cadherin (CADH1) that bind to β-catenin (CTNB1) and α-catenin (CTNA1). Alpha-catenin links the E-cadherin/β-catenin complex to the actin cytoskeleton, acting as a bridge to connect cell adhesion molecules to the cytoskeleton, which is crucial for the mechanical stability of the cell layer [9].

Vitamin D plays a crucial role in skin health, influencing processes such as keratinocyte proliferation, differentiation, and immune regulation [10,11,12,13]. Its actions in the skin are mediated by the vitamin D receptor (VDR) [14]. Vitamin D, whether synthesized in the skin or obtained from the diet, is first hydroxylated in the liver to form 25-hydroxyvitamin D (25(OH)D), the main circulating form used to evaluate vitamin D status [15,16].

The skin expresses the enzyme CYP27B1, which converts 25(OH)D into its active form, 1,25-dihydroxyvitamin D (1,25(OH)_2_D_3_), and the enzyme CYP24A1, which is responsible for degradation of the active form [15]. Vitamin D also plays a role in the skin’s innate immune defense by regulating the production of antimicrobial peptides (AMPs), such as cathelicidin (CAMP) [14]. CAMP, produced as the inactive peptide hCAP18, is cleaved into the active form LL-37, which protects against microbial invasion by disrupting bacterial membranes. Its expression increases after skin injury, partly through enhanced CYP27B1 activity, which boosts local synthesis of active vitamin D, promoting cathelicidin production [17]. This mechanism enhances the skin’s ability to respond to infection and maintain barrier function.

Haptoglobin (HPT) is a protein primarily synthesized in the liver but also in tissues such as the skin [18], where it plays roles beyond hemoglobin scavenging, including acting as an anti-inflammatory agent, an antioxidant, and a T-cell immunosuppressor [19,20]. Given its involvement in immune regulation, HPT may influence inflammatory processes in AD, similar to its proposed role in other skin diseases like psoriasis [21]. These properties suggest that haptoglobin could contribute to immune dysregulation in AD.

Currently, no data are available on the expression of proteins involved in tight junctions, the epithelial barrier, vitamin D metabolism, and the immune response and inflammation in peri-lesional (PL) and intra-lesional (IL) biopsies of AD patients. Skin biopsies remain the preferred method for analyzing immune and barrier characteristics in AD [22].

Previous studies have shown significant molecular and cellular differences between lesional and non-lesional AD skin, including in inflammatory cell infiltration and protein expression [22,23,24,25]. We recently identified associations between VDR and tight junction proteins, showing a negative relationship between serum 25(OH)D levels and zonulin-1 expression in AD patients [26]. The aim of this cross-sectional exploratory study was to compare the expression of a range proteins involved in tight junctions, the epithelial barrier, vitamin D metabolism, and the immune response and inflammation from IL and PL AD biopsies to further explore their roles in the disease’s pathogenesis and their clinical significance.

## 2. Results

### 2.1. Patient Characteristics

A total of 22 adult participants with AD (59.1% male) were included (Table 1). The majority of the patients (N = 17; 77.3%) had moderate-to-severe disease, characterized by an EASI score ≥16 or <16 with face or hand involvement (Table 1). Most patients presented with generalized AD (N = 8; 36.4%), and childhood onset was predominant, as reported by 59.1% (N = 13) of the patients. Comorbid asthma and rhinoconjunctivitis were observed in 9.1% (N = 2) and 31.8% (N = 7) of the patients, respectively, while both conditions coexisted in a subset of patients. Skin prick tests (SPTs) were positive in 54.5% (N = 12) of the patients, and total IgE levels were ≥100 IU/mL in 50.0% (N = 11). Additionally, 72.7% (N = 16) of the patients had insufficient serum vitamin D levels (<30 ng/mL 25(OH)D).

### 2.2. Comparison of Protein Concentrations in Intra- and Peri-Lesional Biopsies

Protein concentrations in PL and IL samples from each patient were compared (Supplementary Table S1). The mean total protein concentration was higher in IL samples (6.8 ± 1.3 mg/mL) than in PL samples (4.0 ± 1.0 mg/mL, *p* < 0.001) (Supplementary Figure S1a). For each patient, the protein concentration was also consistently higher in IL samples than in PL samples (Supplementary Figure S1b), equating to a mean percentage increase of 77.7 ± 50.1% in IL samples.

**Figure 1:**
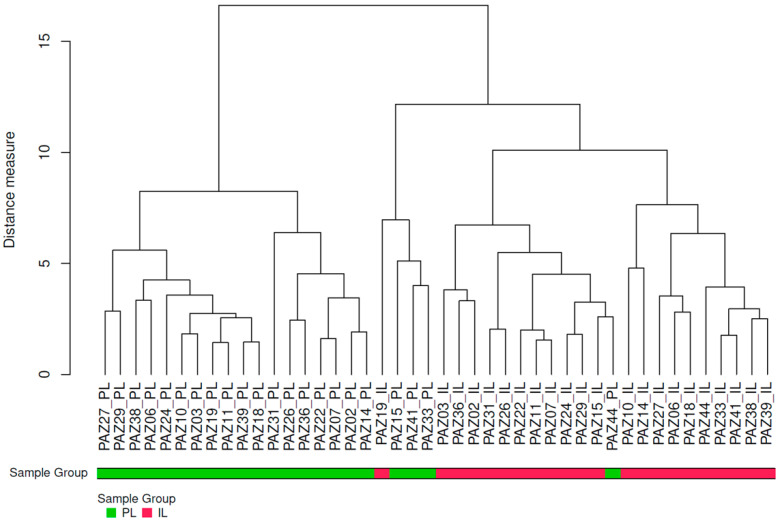
Dendrogram of hierarchical clustering of protein expression profiles from intra-lesional (IL) and peri-lesional (PL) biopsy samples using the complete protein data set. The dendrogram illustrates the clustering of all proteins extracted from PL and IL samples. The *x*-axis represents individual samples, while the *y*-axis indicates the distance measure used for clustering. PAZ = patient (identification code assigned to each patient).

Hierarchical clustering analysis of protein expression profiles from PL and IL biopsy samples by dendrogram revealed two main clusters: one containing only PL samples (denoted in red) and the other encompassing all IL samples (denoted in green) and a few PL samples (Figure 1). This primary clustering was further subdivided into two distinct subclusters within each main group, indicating molecular differences within the main sample categories. The unique clustering of PL samples suggests a specific set of proteins associated with the PL environment, reflecting the biological processes in the tissue surrounding lesions. In contrast, IL samples exhibited a separate clustering pattern, identifying proteins potentially involved in the IL microenvironment.

### 2.3. Differential Protein Expression

**Figure 2:**
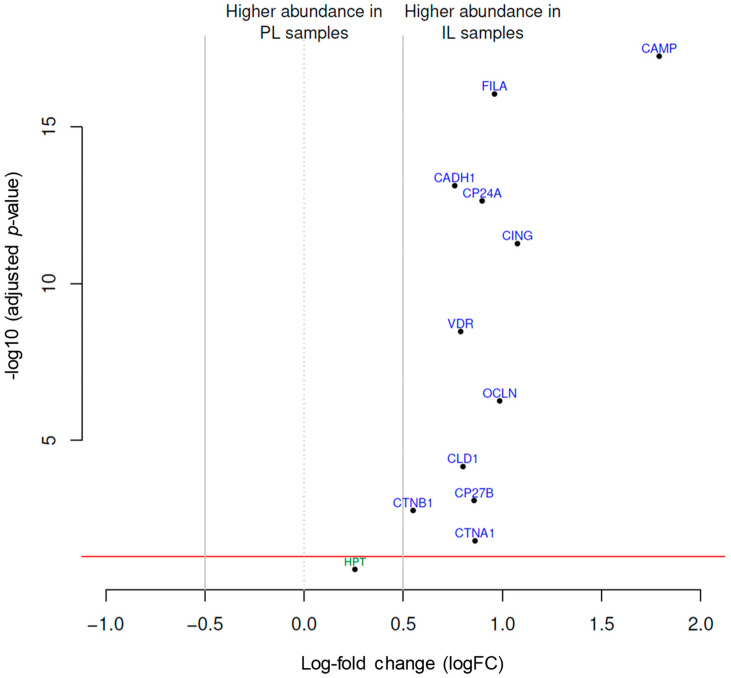
Distribution pattern of protein expression in IL and PL samples from patients with AD. The volcano plot presents the adjusted *p*-values and corresponding log-fold changes (logFCs). The significance level of an adjusted *p*-value = 0.05 is indicated by a horizontal red line. Vertical lines denote the logFC cutoffs. Proteins with a positive logFC are more abundant in IL samples, whereas those with a negative logFC are more abundant in PL samples. Proteins with |logFC| > 0.5 and a significant adj. *p*-value are considered differential and are displayed with blue names. Proteins with |logFC| > 0 but not reaching the significance threshold are indicated with green names. PL represents peri-lesional biopsies, and IL corresponds to intra-lesional biopsies. AD = atopic dermatitis; CAMP = cathelicidin; CADH1 = cadherin-1; CING = cingulin; CLD1 = claudin-1; CTNA1 = alpha-catenin; CTNB1 = beta-catenin; CYP24A1 = cytochrome P450 family 24 subfamily A member 1; CYP27B1 = cytochrome P450 family 27 subfamily B member 1; FILA = filaggrin; HPT = haptoglobin; OCLN = occludin.

Analysis of protein expression revealed significant differences among several proteins in IL samples compared to PL samples (Figure 2, Table 2, and Supplementary Figure S2). CAMP revealed the highest log-fold change (logFC = 1.79) and an average expression (AveExp) of 12.82 (adjusted *p*-value = 5.5 × 10^−18^) (Table 2). Other proteins such as cingulin, (logFC = 1.08, AveExp = 10.11, adj.p = 5.3 × 10^−12^), occludin-1 (logFC = 0.99, AveExp = 10.48, adj.p = 5.5 × 10^−7^), and filaggrin (logFC = 0.96, AveExp = 10.46, adj.p = 8.9 × 10^−17^) also demonstrated marked (~2-fold increase) differences (Table 2).

**Figure 3:**
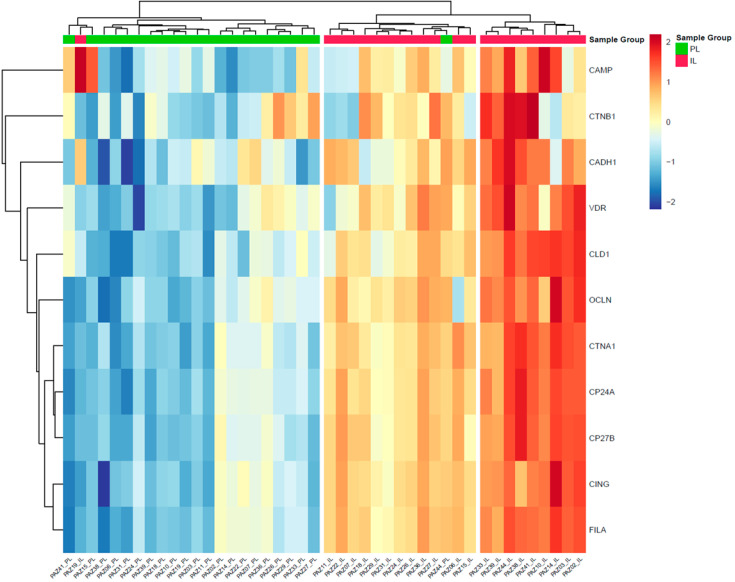
Heatmap depicting protein expression profiles in intra-lesional (IL) and peri-lesional (PL) skin samples from patients with AD. The heatmap displays the log-fold change (logFC) in protein expression levels, with red hues indicating higher expression and blue hues indicating lower expression. AD = atopic dermatitis; CAMP = cathelicidin; CADH1 = cadherin-1; CING = cingulin; CLD1 = claudin-1; CTNA1 = alpha-catenin; CTNB1 = beta-catenin; CYP24A1 = cytochrome P450 family 24 subfamily A member 1; CYP27B1 = cytochrome P450 family 27 subfamily B member 1; FILA = filaggrin; OCLN = occludin; PAZ = patient (identification code assigned to each patient); VDR = vitamin D receptor.

Analysis of protein expression profiles using heatmapping revealed distinct patterns between PL and IL skin samples from individuals with AD (Figure 3). Similarly, the clustering of proteins showed patterns of co-regulation, with some proteins such as CAMP, VDR, and beta-catenin forming groups that are more highly expressed in IL samples, while others clustered together due to their lower expression in IL regions.

### 2.4. Associations Between Clinical Characteristics and Protein Expression in Intra- and Peri-Lesional Areas

Sub-analysis of different patient characteristics revealed significant differences in levels of protein expression that were predominantly derived from IL areas (Table 3). The levels of beta-catenin expression were found to be significantly higher in females than in males (*p* = 0.034). Patients with 25(OH)D levels < 30 ng/mL showed higher expression of CYP24A (*p* = 0.054), alpha-catenin (*p* = 0.043), and haptoglobin (*p* = 0.033) than those with 25(OH)D levels ≥ 30 ng/mL. Similarly, patients with serum 25(OH)D < 20 ng/mL had significantly elevated haptoglobin expression (*p* = 0.021). In SPT-positive patients, VDR (*p* = 0.006), CYP24A (*p* = 0.025), CYP27B (*p* = 0.022), filaggrin (*p* = 0.027), alpha-catenin (*p* = 0.022), and cingulin (*p* = 0.052) expression levels were significantly higher than those in SPT-negative patients. Furthermore, patients with IgE levels ≥ 100 IU/mL exhibited increased levels of VDR (*p* = 0.007), occludin-1 (*p* = 0.011), and beta-catenin (*p* = 0.041) expression. EASI scores ≥ 16 were also found to be associated with higher expression levels of CYP24A (*p* = 0.024), CYP27B (*p* = 0.044), filaggrin (*p* = 0.027), occludin-1 (*p* = 0.049), and claudin-1 (*p* = 0.052).

In contrast, in PL areas, although fewer significant findings emerged (Supplementary Table S2), haptoglobin expression was found to be higher in patients with 25(OH)D < 20 ng/mL (*p* = 0.034) and in SPT-positive patients (*p* = 0.016). Alpha-catenin expression was also higher in patients with IgE levels ≥ 100 IU/mL (*p* = 0.035).

Multivariate regression analysis confirmed several significant associations identified by sub-analysis (Table 4). CAMP expression was negatively associated with a childhood AD onset (β = −0.26, *p* = 0.0396), while claudin-1 (β = 2.06, *p* = 0.048) and alpha-catenin (β = −4.16, *p* = 0.016) were linked to the flexural phenotype. Haptoglobin expression correlated negatively with asthma/rhinoconjunctivitis (β = −0.39, *p* = 0.05), and low 25(OH)D levels (<30 ng/mL) were associated with claudin-1 (β = −2.04, *p* = 0.0077) and occludin-1 (β = 0.43, *p* = 0.002). In skin prick test-positive patients, claudin-1 (β= −1.93, *p* = 0.046), CAMP (β = 0.26, *p* = 0.027), and occludin-1 (β = −0.33, *p* = 0.044) expression showed significant associations with the phenotype.

In PL areas (Supplementary Table S3), younger age was associated with lower cingulin levels (β = −1.62, *p* = 0.0061), and higher EASI scores correlated with increased alpha-catenin expression (β = 3.99, *p* = 0.026).

## 3. Discussion

In this cross-sectional exploratory study, we observed significant differences in protein concentrations and expression levels between IL and PL biopsies from patients with AD. Cluster analysis revealed clear, distinct profiles for protein expression between IL and PL samples, and the differential expression of proteins involved in the epithelial barrier, vitamin D metabolism, and immune response pathways was strongly associated with disease severity. Higher levels of protein expression in IL areas correlated with a positive SPT, elevated IgG levels, and EASI scores ≥16. Multivariate regression demonstrated a negative correlation between low vitamin D levels and the expression of specific TJ proteins.

TJs are recognized as a key component of the complex epidermal barrier and are located in the stratum granulosum, where they regulate the passage of ions and molecules [27,28,29]. Claudin-1, occludin-1, and cingulin are among the primary proteins forming these junctions [6]. We observed increased expression levels of these proteins in IL samples compared to PL samples. However, previous studies have reported conflicting data regarding their expression.

In the literature to date, some studies report wide variability in claudin-1 levels in healthy skin [4], while others have noted decreased claudin-1 expression in lesional areas compared to non-lesional and normal skin [30,31,32].

The finding that levels of occludin-1 expression were increased in peri-lesional skin align with those of Bergmann et al., who also observed upregulation in lesional skin [31]. In contrast, Gruber et al. [30] did not observe any significant alterations in occludin levels. To our knowledge, differences in cingulin protein expression between IL and PL skin in AD have not been reported previously. Similarly, proteins involved in adherens junctions (cadherin-1, beta-catenin, and alpha-catenin) were overexpressed in IL samples compared to PL samples in our study, although there are limited reports of such findings in the literature. Nelson et al. [33] observed unaltered cadherin-1 expression in a small set of AD lesions. Filaggrin, another key protein involved in the maintenance of skin barrier integrity [5], was among the most highly expressed proteins observed in IL samples compared to PL samples in our study. These findings are inconsistent with those of several other studies that report reduced filaggrin expression in lesional skin [34,35,36]. These differences between our findings and those of other studies may be explained by differences in the samples that were compared. Our analysis compared IL (intra-lesional skin) and PL (peri-lesional skin adjacent to a lesion) biopsies, while other studies generally compared lesional skin to non-lesional skin, defined as skin far from a lesion. PL skin, as noted by Knor et al. [37], exhibits distinct characteristics, such as altered pH and hydration, compared to non-lesional skin, potentially explaining the observed differences in protein expression. In addition, the cluster analysis in our study also revealed a clear and distinct map discriminating between IL and PL samples, reflecting unique differences in molecular diversity between these two sample types.

It could also be hypothesized that the increased expression of skin barrier proteins in IL regions may represent a compensatory response aimed at maintaining barrier integrity amidst the heightened turnover associated with inflammation in AD. Alternatively, these increased levels of protein expression may reflect an attempt to repair the damaged barrier, even though ongoing dysfunction likely perpetuates inflammation. Additionally, the chronic inflammation observed in AD may be related to increased levels of interleukin (IL)-4, IL-13, and IL-31, which can cause alterations in both the structures and expression levels of occludin-1, cingulin, and claudin-1 with the aim of re-establishing barrier function, although with limited efficacy [38,39,40]. Furthermore, overexpression of these proteins in IL areas does not necessarily guarantee functional barrier integrity. Elevated levels may instead represent a compensatory mechanism counteracting the loss of barrier function. It is worth noting that filaggrin deficiency, for example, does not directly affect TJ-related barrier integrity [4].

Studies in filaggrin-knockout mice showed no alterations in TJ morphology, expression, or function, suggesting that underlying inflammation, rather than protein loss, drives TJ dysfunction [41]. This dysfunction leads to SC damage and increased permeability to bacterial and allergenic molecules, creating a vicious cycle of barrier dysfunction and skin inflammation [41].

Taken together, these observations may also have future therapeutic implications, highlighting the importance of restoring the skin barrier through topical (emollients and moisturizers) and systemic treatments, supporting tight junctions, and improving skin integrity.

We observed higher expression levels of VDR and vitamin D metabolism-associated enzymes in IL areas than in PL areas. Similarly, Weise et al. [42] reported increased VDR and CYP24A1 expression in the lesional and non-lesional skin of AD patients compared to healthy controls. Moreover, we found higher levels of CAMP in IL areas than in PL areas, which aligns with its role in responding to barrier disruption or infection [14]. CAMP expression increases following skin injury and is linked to enhanced CYP27B1 expression and local synthesis of active vitamin D [14]. This increased expression may reflect an amplified immune response aimed at controlling inflammation, although it may also contribute to chronic underlying inflammation in AD. The direct regulation of CAMP by the 1,25(OH)_2_D_3_–VDR complex [43], as well as vitamin D’s broader role in modulating CAMP synthesis, highlights its involvement in skin defense and supports the overexpression of both VDR and CAMP in IL samples in our study.

We also identified some specific associations between protein expression levels and AD disease severity. Higher levels of skin barrier- and vitamin D metabolism-associated proteins were observed in patients with a positive SPT, IgE levels ≥ 100 IU/mL, and EASI scores ≥ 16, which are indicative of moderate–severe eczema [44]. Correspondingly, we found fewer associations between these clinical data and protein expression levels in PL regions, suggesting that these correlations do not exist where the disease is mild in severity or absent. In line with our findings, Grieco et al. [26] previously reported a correlation between TJ protein expression and a positive SPT (OR: 8.23, 95% CI 1.04–65.5, *p* = 0.046). Additionally, in children with AD, oral 1,25(OH)_2_D_3_ supplementation was associated with improved vitamin D status and AD severity and increased VDR expression in lesional skin but not in non-lesional skin [45].

We also observed a negative correlation between low vitamin D levels and the expression of claudin-1 by multivariate regression. In ulcerative colitis, an inflammatory disease characterized by gut barrier dysfunction, claudin-1 protein expression was found to be upregulated [46]. In patients with ulcerative colitis, treatment with 1,25(OH)_2_D_3_ decreased claudin-1 protein levels in both inflamed and non-inflamed tracts [46]. This suggests that increasing vitamin D levels could reduce the expression of some specific TJ proteins, potentially lowering inflammation and the compensatory mechanisms that lead to their increased expression. Taken together, these findings suggest that restoring vitamin D levels could be a promising therapeutic strategy for modulating TJ protein expression and enhancing skin barrier function in AD. Vitamin D plays a crucial role in regulating antimicrobial peptides (e.g., CAMP) and the VDR pathway, which may help alleviate chronic inflammation. Topical or systemic vitamin D supplementation, in combination with creams, could strengthen the skin’s barrier, reducing inflammation and promoting healing. Moreover, improving vitamin D status may help regulate the compensatory upregulation of TJ proteins, potentially offering a more targeted approach. This approach could also lead to more personalized treatment options by addressing the underlying molecular mechanisms contributing to disease severity. By understanding how specific proteins influence AD, dermatologists can develop more effective strategies for managing the condition and improving patient outcomes.

### Limitations

There are some limitations of this study that should be noted. The small sample size limits the generalizability of the findings. While the small sample size (N = 22) may raise concerns, the design and methods used compensate for this limitation. This study adopted an intra-patient design. This approach reduces variability caused by differences between patients and often requires fewer participants to achieve statistically meaningful results.

Antibody microarrays are a highly accurate and validated technique used to measure protein expression, ensuring the reliability of results. Given the large differences in expression observed for some proteins between IL and PL areas, a larger sample size may not have been warranted. There are some potential confounding variables that need to be mentioned. First, while we compared IL and PL areas, distant non-lesional skin was not included as a control or obtained from a population without AD for ethical reasons. However, PL biopsies were always taken at a distance of 3 cm from the margin of the lesion, where no obvious lesions were visible and maintained characteristics consistent with those of more distant skin. Second, although we excluded patients with underlying conditions, other inflammatory diseases, and recent corticosteroid use, which could impact vitamin D metabolism and potentially tight junction protein expression, we did not exclude patients who may have been receiving or had recently received other treatments, which may have impacted the results observed. Third, a substantial proportion of the patients presented with moderate-to-severe disease; therefore, the study population may not have been fully representative of the overall AD population in the real-life setting. Despite this, the focus on more severe cases was critical for identifying significant molecular alterations. Future studies should aim to include more heterogeneous patient cohorts, larger sample sizes, and populations with greater variation in disease severity to generalize these findings. Last, the cross-sectional design prevents drawing conclusions about causal links between protein expression and AD severity. Additionally, we did not fully control for variability in sample collection and environmental factors such as seasonal changes or treatments.

## 4. Materials and Methods

### 4.1. Study Design and Patients

This was a monocentric, cross-sectional, exploratory study involving 22 adult patients with acute-phase atopic dermatitis (AD), a subset of a cohort previously enrolled at the Dermatology Clinic of Policlinico Umberto I, Sapienza University of Rome, Italy [26]. Inclusion and exclusion criteria and patient characteristics are described elsewhere [26].

Briefly, this study included males and females aged ≥18 years with mild or moderate–severe atopic dermatitis. The exclusion criteria were other inflammatory/autoimmune skin diseases, calcium/bone metabolism issues, recent corticosteroid use, severe diseases, certain infections, organ transplants, dementia, psychosis, substance abuse, pregnancy, anticoagulation, and recent sun exposure. No dietary restrictions were imposed. During the initial screening, data collection included demographic information (gender, age, BMI), medical and pharmacological history, age at AD onset, AD phenotype, disease severity (assessed by the EASI [47,48]), and the presence of comorbid conditions such as asthma or allergic rhinitis.

Blood samples were also collected at this stage. Within 10 days of the screening visit, patients returned for further evaluation, including skin biopsy for protein expression analysis. This study was approved by the head of the Unit of the Dermatological Clinic of the Policlinico Umberto I, University of Rome, La Sapienza and conducted in accordance with the Declaration of Helsinki and adhered to Good Clinical Practice (GCP) standards, International Council for Harmonisation guidelines, and applicable national regulations. The study protocol was approved by the ethics committee (identification code: DERM/AT/01).

### 4.2. Biopsy Sampling

**Figure 4:**
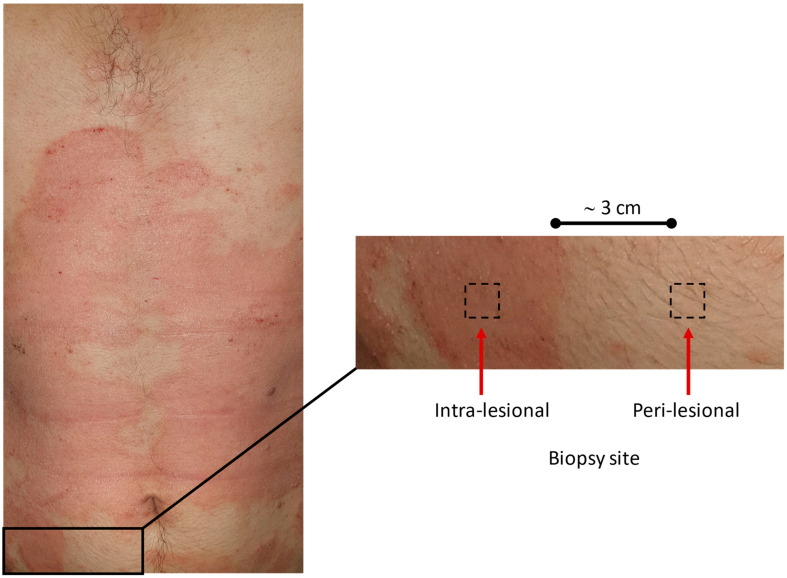
Image of the thoracic region and abdomen of a male patient with atopic dermatitis. Large erythematous, scaly plaques covering the central trunk are visible. The skin also appears excoriated in some areas. The two regions highlighted with red arrows indicate the sites of intra-lesional and peri-lesional biopsies. The biopsied areas appear slightly erythematous and textured, consistent with the surrounding dermatitis-affected skin. Peri-lesional biopsies were taken approximately 3 cm from the edge of the lesion.

At the initial visit, all AD patients underwent a single biopsy of a skin lesion (intra-lesional, IL) and another biopsy in the peri-lesional region (PL) approximately 3 cm from the margin of the lesion (Figure 4). After antiseptic preparation using povidone iodine, local anesthesia was administered with 2% mepivacaine. A tissue sample measuring up to 10 × 5 mm was excised. The biopsy site was then closed with 1–3 nylon (3.0) sutures, and a medicated bandage was applied. Patients were prescribed antibiotic prophylaxis consisting of 1 g amoxicillin combined with clavulanic acid taken every 12 h for five days. Sutures were removed between 7- and 15-days post-biopsy.

### 4.3. Protein Extraction

Protein extraction was performed from 44 skin samples using scioExtract buffer (Sciomics GmbH, 69151 Neckargemünd, Germany) according to the manufacturer’s standard operating procedures. After extraction and quality control of the samples, protein concentrations were measured using a standard bicinchoninic acid assay. A reference sample was prepared by pooling equal volumes from each individual sample, and protein concentrations were calculated for each sample group.

### 4.4. Sample Labeling and Data Analysis

For protein labeling, the samples were adjusted to a specific concentration and incubated with scioDye 2 (Sciomics GmbH) for two hours. The reference sample was labeled with scioDye 1 (Sciomics GmbH). Following labeling, the reaction was stopped, and the buffer was exchanged for phosphate-buffered saline (PBS). Labeled protein samples were then stored at −20 °C until further analysis. Samples were analyzed using a dual-color approach on 44 custom antibody microarrays (Sciomics GmbH) targeting 12 different proteins (Table 5), with each antibody represented in 4 replicates. Arrays were blocked with scioBlock (Sciomics GmbH) using a Hybstation 4800 (Tecan, 5082 Grödig, Austria), and the samples were incubated competitively with the reference sample. After three-hour incubation, slides were washed with 1 × PBSTT, rinsed with 0.1 × PBS and water, and dried with nitrogen. Data acquisition was performed using a Powerscanner (Tecan, Austria) with consistent laser power and photomultiplier tube settings. Spot segmentation was performed using GenePix Pro 6.0 (Molecular Devices, Union City, CA 94587, USA). Raw data were analyzed using the LIMMA package in R-Bioconductor (version 3.34.0, Open Source Software) with median signal intensities.

### 4.5. Statistical Analysis

The clinical characteristics of the patients are presented as numbers and percentages. After confirming a normal distribution by the Kolmogorov–Smirnov test, protein concentrations in IL and PL samples were compared using paired *t*-tests to assess differences in means.

Hierarchical clustering analysis was employed to identify distinct patterns in protein expression profiles between IL and PL samples. For protein analysis, statistical analysis involved fitting a one-factorial linear model, and results were derived from two-sided *t*-tests or F-tests based on moderated statistics. *p*-values were adjusted for multiple comparisons using the false discovery rate method by the Benjamini and Hochberg procedure [50]. Proteins were considered differentially expressed if |logFC| was >0.5 and the adjusted *p*-value was <0.05. Changes in protein abundance between samples or groups are reported as log-fold changes (logFCs), where a logFC of 1 indicates a two-fold increase compared to the control group, and a logFC of −1 indicates a two-fold decrease.

Sub-analysis was performed to evaluate potential associations between demographic factors and vitamin D levels in relation to protein expression using unpaired *t*-tests for normally distributed variables and the chi-squared test for categorical variables. Multivariate regression was used to evaluate associations between protein expression and clinical features in AD patients. The results are reported as correlation coefficients and 95% confidence intervals (CI). A *p*-value < 0.05 was considered statistically significant. Statistical analysis was performed using MedCalc Software (version 23.0.2, Broekstraat, 9030 Mariakerke, Belgium).

## 5. Conclusions

Our findings suggest that the molecular landscape of IL areas in AD patients differs from that of PL regions, showing greater inflammation and a stronger compensatory response to skin barrier dysfunction. Significant associations between protein expression in IL areas and clinical markers, IgE levels, and EASI scores suggest that these proteins may serve as biomarkers for AD progression. The negative correlation between vitamin D levels and TJ protein expression highlights the complex interplay between vitamin D metabolism and skin barrier integrity in AD. Larger cohorts and longitudinal studies are needed to validate these results. Restoring vitamin D levels may improve skin barrier function in AD by regulating TJ proteins and reducing inflammation. Topical or systemic vitamin D supplementation, alongside emollients, could enhance barrier integrity, alleviate symptoms, and promote healing. This approach may offer a more targeted, personalized treatment by addressing underlying disease mechanisms and improving patient outcomes. Future longitudinal studies will facilitate exploration of the causality of specific TJ proteins and proteins involved in vitamin D metabolism in the pathogenesis of AD and the roles of these proteins as biomarkers for potential therapeutic targets.

## Figures and Tables

**Table 1:** Clinical characteristics of the AD patients (N = 22).

Characteristic	N (%)
Gender, n (%)	
Male	13 (59.1)
Female	9 (40.9)
Age	
<60 years	19 (86.4)
≥60 years	3 (13.6)
EASI score	
Mild (EASI < 16)	5 (22.7)
Moderate-to-severe (EASI ≥ 16 or <16 with face/hand involvement)	17 (77.3)
Phenotype (localization), n (%)	
Generalized	8 (36.4)
Head/neck	7 (31.8)
Flexural sites	6 (27.3)
Hands	1 (4.5)
Age at disease onset, n (%)	
Childhood	13 (59.1)
Adulthood	9 (40.9)
Asthma, n (%)	
Present	2 (9.1)
Absent	20 (90.9)
Rhinoconjunctivitis, n (%)	
Present	7 (31.8)
Absent	15 (68.2)
Skin prick test, n (%)	
Positive	12 (54.5)
Negative	10 (45.5)
Total IgE (IU/mL), n (%)	
≥100 IU/mL	11 (50.0)
<100 IU/mL	11 (50.0)
25(OH)D vitamin D	
≥30 ng/mL	6 (27.3)
<30 ng/mL	16 (72.7)

AD = atopic dermatitis; EASI = Eczema Area and Severity Index. Data are presented as numbers and percentages.

**Table 2:** Proteins with differential abundance in IL samples and PL samples.

Protein	Antibody ID	Uniprot Entry *	HGNC	LogFC	AveExp	Adjusted *p*-Value
CAMP	ab1184	P49913	CAMP	1.79	12.82	5.50 × 10^−18^
CING	ab1193	Q9P2M7	CGN	1.08	10.11	5.30 × 10^−12^
OCLN	ab1169	Q16625	OCLN	0.99	10.48	5.50 × 10^−7^
FILA	ab1199	P20930	FLG	0.96	10.46	8.90 × 10^−17^
CP24A	ab1156	Q07973	CYP24A1	0.9	10.82	2.30 × 10^−13^
CTNA1	ab1187	P35221	CTNNA1	0.86	10.66	0.016
CP27B	ab1185	O15528	CYP27B1	0.86	10.7	8.30 × 10^−4^
CLD1	ab1158	O95832	CLDN1	0.8	10.35	6.90 × 10^−5^
VDR	ab1080	P11473	VDR	0.79	11.12	3.40 × 10^−9^
CADH1	ab1182	P12830	CDH1	0.76	14.71	7.50 × 10^−14^
CTNB1	ab1183	P35222	CTNNB1	0.55	13.24	0.0017
HPT	ab1194	P00738	HP	0.26	14.77	0.13

Changes in protein abundance between samples or groups are reported as log-fold changes (logFCs), where a logFC of 1 indicates a two-fold increase compared to that in the control group, and a logFC of −1 indicates a two-fold decrease. AveExp = average expression; CAMP = cathelicidin; CADH1 = cadherin-1; CING = cingulin; CLD1 = claudin-1; CTNA1 = alpha-catenin; CTNB1 = beta-catenin; CYP24A1 = cytochrome P450 family 24 subfamily A member 1; CYP27B1 = cytochrome P450 family 27 subfamily B member 1; FC = fold change; FILA = filaggrin; HGNC = HUGO Gene Nomenclature Committee; HPT = haptoglobin; IL = intra-lesional biopsies, OCLN = occludin; PL = peri-lesional biopsies; VDR = vitamin D receptor. * https://www.uniprot.org/uniprotkb?query=* (accessed on 19 October 2021).

**Table 3:** Sub-analysis of protein expression levels and clinical features in intra-lesional areas of patients with atopic dermatitis.

			Vitamin D Metabolism			Epithelial Barrier							Immune Response and Inflammation	
		n	VDR	CYP24A1	CPY27B1	FILA	CLD1	OCLN	CADH1	CTNB1	CTNA1	CING	HPT	CAMP
Gender	Male	13	0.5(0.5)	0.8(0.4)	0.9(0.4)	0.8(0.5)	0.7(0.4)	0.6(0.7)	0.7(0.3)	0.4(0.6)	0.9(0.4)	0.9(0.6)	0.4(0.9)	0.9(1.2)
	Female	9	0.5(0.6)	0.8(0.4)	0.9(0.4)	0.8(0.3)	0.7(0.4)	0.8(0.3)	0.9(0.5)	1(0.7)	0.9(0.4)	0.9(0.3)	0.9(0.9)	1.2(1.2)
	*p*-value		0.343	0.366	0.392	0.414	0.477	0.275	0.100	**0.034**	0.469	0.454	0.082	0.278
Localization	Not flexural	16	0.5(0.5)	0.9(0.3)	0.9(0.3)	0.8(0.3)	0.7(0.4)	0.7(0.5)	0.8(0.4)	0.7(0.8)	1(0.3)	0.9(0.4)	0.8(0.8)	1.1(1.1)
	Flexural	6	0.5(0.6)	0.7(0.6)	0.8(0.5)	0.7(0.6)	0.7(0.5)	0.6(0.8)	0.7(0.3)	0.4(0.6)	0.8(0.5)	0.8(0.8)	0.2(1.1)	0.8(1.3)
	*p*-value		0.431	0.215	0.287	0.233	0.363	0.289	0.262	0.156	0.147	0.264	0.087	0.342
25(OH)D Level	≥30 ng/mL	6	0.4(0.6)	0.6(0.5)	0.7(0.5)	0.6(0.6)	0.6(0.5)	0.4(0.7)	0.6(0.4)	0.4(0.5)	0.7(0.5)	0.6(0.8)	0(1.3)	1.6(1.2)
	<30 ng/mL	16	0.5(0.5)	0.9(0.3)	0.9(0.3)	0.9(0.3)	0.8(0.3)	0.8(0.5)	0.9(0.4)	0.7(0.8)	1(0.3)	1(0.3)	0.8(0.7)	0.8(1.1)
	*p*-value		0.241	**0.054**	0.065	0.072	0.215	0.068	0.101	0.178	**0.043**	0.075	**0.033**	0.078
	≥20 ng/mL	16	0.5(0.6)	0.8(0.4)	0.8(0.4)	0.8(0.5)	0.7(0.4)	0.7(0.6)	0.8(0.4)	0.5(0.7)	0.9(0.4)	0.9(0.6)	0.4(0.9)	1.1(1.2)
	<20 ng/mL	6	0.4(0.4)	0.9(0.3)	1(0.3)	0.9(0.2)	0.8(0.3)	0.7(0.5)	0.8(0.3)	1(0.8)	1(0.3)	0.9(0.2)	1.2(0.6)	0.7(1)
	*p*-value		0.326	0.179	0.152	0.307	0.392	0.446	0.491	0.094	0.167	0.466	**0.021**	0.209
Skin Prick Test	Negative	10	0.2(0.5)	0.6(0.4)	0.7(0.4)	0.6(0.5)	0.6(0.4)	0.5(0.5)	0.7(0.3)	0.4(0.6)	0.7(0.4)	0.7(0.6)	0.9(0.6)	0.7(1.4)
	Positive	12	0.7(0.4)	1(0.3)	1(0.3)	0.9(0.3)	0.8(0.3)	0.8(0.5)	0.9(0.4)	0.9(0.8)	1.1(0.3)	1(0.4)	0.4(1.1)	1.3(0.9)
	*p*-value		**0.006**	**0.025**	**0.022**	**0.027**	0.061	0.065	0.122	0.060	**0.022**	**0.052**	0.090	0.118
IgE Level	<100 IU/mL	11	0.2(0.5)	0.7(0.4)	0.8(0.4)	0.7(0.4)	0.6(0.4)	0.4(0.6)	0.8(0.3)	0.4(0.8)	0.8(0.4)	0.7(0.5)	0.8(0.7)	1(1.4)
	≥100 IU/mL	11	0.8(0.4)	0.9(0.4)	1(0.4)	0.9(0.4)	0.8(0.4)	0.9(0.4)	0.8(0.5)	0.9(0.6)	1(0.4)	1(0.5)	0.4(1.1)	1(0.9)
	*p*-value		**0.007**	0.113	0.133	0.120	0.168	**0.011**	0.393	**0.041**	0.143	0.106	0.142	0.440
EASI Score	<16	5	0.2(0.5)	0.5(0.5)	0.6(0.5)	0.5(0.5)	0.5(0.3)	0.3(0.7)	0.6(0.3)	0.5(0.8)	0.6(0.4)	0.5(0.7)	0.7(1.4)	1(1.4)
	≥16	17	0.6(0.5)	0.9(0.3)	0.9(0.3)	0.9(0.3)	0.8(0.4)	0.8(0.5)	0.9(0.4)	0.7(0.7)	1(0.3)	1(0.4)	0.6(0.8)	1(1.1)
	*p*-value		0.111	**0.024**	**0.044**	**0.027**	0.059	**0.049**	**0.052**	0.294	**0.013**	**0.025**	0.393	0.490

Data are presented as the levels of protein expression involved in vitamin D metabolism, the epithelial barrier, and the immune response and inflammation stratified by gender, localization, 25(OH)D level, skin prick test result, IgE level, and EASI score (Eczema Area and Severity Index). *p*-values < 0.05 are highlighted in bold text. Data are expressed as the mean log2 (sample/reference) expression level and (SD). CAMP = cathelicidin; CADH1 = cadherin-1; CING = cingulin; CLD1 = claudin-1; CTNA1 = alpha-catenin; CTNB1 = beta-catenin; CYP24A1 = cytochrome P450 family 24 subfamily A member 1; CYP27B1 = cytochrome P450 family 27 subfamily B member 1; FILA = filaggrin; HPT = haptoglobin; OCLN = occludin; VDR = vitamin D receptor.

**Table 4:** Multivariate regression evaluating the associations between protein expression levels in intra-lesional areas and clinical parameters.

Vitamin D Metabolism					Epithelial Barrier							Immune Response and Inflammation	
		CYP24A1	CYP27B1	VDR	OCLN	CING	CLD1	FILA	CADH1	CTNA1	CTNB1	CAMP	HPT
Age at Onset (Childhood)	β (95% CI)	−0.56 (−6.28,5.16)	0.46 (−2.63,3.55)	0.34 (−0.74,1.42)	−0.52 (−1.51,0.48)	−0.54 (−3.36,2.29)	1.14 (−0.99,3.26)	−1.34 (−8.62,5.93)	−0.04 (−0.74,0.65)	2.06 (−1.26,5.38)	−0.04 (−0.70,0.62)	−0.26 (−0.51,−0.02)	−0.05 (−0.41,0.31)
	S.E.	2.53	1.37	0.48	0.44	1.25	0.94	3.22	0.31	1.47	0.29	0.11	0.16
	T	−0.22	0.34	0.72	−1.17	−0.43	1.21	−0.42	−0.15	1.41	−0.15	−2.40	−0.31
	*p*-value	0.83	0.74	0.49	0.27	0.68	0.26	0.69	0.89	0.19	0.88	**0.039**	0.76
Phenotype (Flexural)	β (95% CI)	3.53 (−1.96,9.03)	2.46 (−0.51,5.43)	0.44 (−0.60,1.47)	−0.85 (−1.81,0.11)	1.37 (−1.35,4.09)	2.06 (0.02,4.10)	−4.56 (−11.55,2.43)	−0.16 (−0.83,0.51)	−4.16 (−7.34,−0.97)	−0.17 (−0.81,0.46)	−0.15 (−0.38,0.09)	−0.19 (−0.54,0.15)
	S.E.	2.43	1.31	0.46	0.42	1.20	0.90	3.09	0.29	1.41	0.28	0.10	0.15
	T	1.45	1.87	0.95	−2.00	1.14	2.28	−1.48	−0.55	−2.95	−0.62	−1.38	−1.28
	*p*-value	*0.18*	*0.094*	*0.37*	*0.077*	*0.28*	***0.048***	*0.17*	*0.60*	***0.016***	*0.55*	*0.19*	*0.23*
Asthma or Rhinoconjunctivitis (Present)	β (95% CI)	−0.76 (−6.95,5.54)	1.24 (−2.11,4.59)	0.23 (−0.94,1.40)	−0.13 (−1.21,0.95)	1.56 (−1.50,4.62)	−0.26 (−2.56,2.04)	−2.68 (−10.56,5.21)	0.42 (−0.33,1.18)	−0.06 (−3.65,3.53)	0.40 (−0.32,1.11)	−0.02 (−0.29,0.25)	−0.39 (−0.77,−0.00)
	S.E.	2.74	1.48	0.52	0.48	1.35	1.02	3.48	0.33	1.59	0.32	0.12	0.17
	T	−0.28	0.84	0.45	−0.27	1.15	−0.25	−0.77	1.28	−0.04	1.26	−0.19	−2.26
	*p*-value	0.79	0.42	0.66	0.79	0.28	0.80	0.46	0.23	0.97	0.24	0.86	**0.05**
Skin Prick Test (Positive)	β (95% CI)	−2.69 (−7.75,2.37)	1.87 (−0.87,4.60)	0.60 (−0.35,1.56)	−0.00 (−0.88,0.88)	−0.23 (−2.73,2.27)	−1.93 (−3.81,−0.05)	0.04 (−6.40,6.48)	0.40 (−0.21,1.02)	2.61 (−0.32,5.55)	0.09 (−0.49,0.68)	0.26 (0.04,0.47)	−0.33 (−0.64,−0.01)
	S.E.	2.24	1.21	0.42	0.39	1.11	0.83	2.85	0.27	1.30	0.26	0.10	0.14
	T	−1.20	1.54	1.43	−0.01	−0.21	−2.32	0.01	1.48	2.01	0.35	2.64	−2.35
	*p*-value	0.26	0.16	0.19	0.99	0.84	**0.046**	0.99	0.17	0.075	0.73	**0.027**	**0.044**
25(OH)D Level (<30 ng/mL)	β (95% CI)	0.97 (−2.68,4.61)	−1.20 (−3.17,0.77)	0.32 (−0.37,1.00)	0.70 (0.07,1.34)	0.53 (−1.27,2.33)	−2.04 (−3.39,−0.69)	−0.91 (−5.54,3.72)	0.26 (−0.18,0.70)	1.81 (−0.30,3.92)	−0.30 (−0.72, 0.12)	−0.06 (−0.22, 0.10)	0.43 (0.20,0.66)
	S.E.	1.61	0.87	0.3	0.28	0.8	0.6	2.05	0.2	0.93	0.19	0.07	0.1
	T	0.6	−1.38	1.04	2.5	0.67	−3.41	−0.44	1.33	1.94	−1.62	−0.88	4.28
	*p*-value	0.56	0.20	0.32	**0.034**	0.52	**0.0077**	0.67	0.22	0.084	0.14	0.40	**0.002**

The table reports only the clinical parameters for which at least one significant association (*p* < 0.05) was found among the analyzed proteins. Coefficients (β), 95% confidence intervals (CIs), and *p*-values are shown for each variable. n.d. represents not determined, S.E. indicates standard error, and T corresponds to the T statistic. CAMP = cathelicidin; CADH1 = cadherin-1; CING = cingulin; CLD1 = claudin-1; CTNA1 = alpha-catenin; CTNB1 = beta-catenin; CYP24A1 = cytochrome. P450 family 24 subfamily A member 1; CYP27B1 = cytochrome P450 family 27 subfamily B member 1; FILA = filaggrin; HPT = haptoglobin; OCLN = occludin; VDR = vitamin D receptor. Statistically significant *p*-values (<0.05) are indicated in bold text.

**Table 5:** Summary of proteins involved in pathways related to tight junctions, the immune response, and vitamin D metabolism, the expression levels of which were investigated in this study.

Pathway	Protein *	Function
Epithelial Barrier	CADH1	Cadherin-1, also known as E-cadherin, is crucial for cell–cell adhesion, maintaining epithelial cell layer integrity, and suppressing cancer cell invasion.
	CTNB1	Beta-catenin, a key component of the Wnt signaling pathway, is involved in cell adhesion and gene transcription regulation.
	OCLN	Occludin is a transmembrane protein that plays a critical role in the formation and maintenance of tight junctions between cells, regulating paracellular permeability.
	CLD1	Claudin-1 is the major tight junction component controlling epithelial permeability and is essential for skin barrier function.
	FILA	Filaggrin is a key protein in the epidermis that helps maintain skin barrier function by aggregating keratin filaments and contributing to the formation of the skin’s outermost layer.
	CING	Cingulin is involved in the organization of tight junctions and interacts with other junctional proteins to regulate cell signaling and barrier function.
	CTNA1	Alpha-catenin links cadherins to the actin cytoskeleton, playing a vital role in cell adhesion and maintaining tissue architecture.
		
Immune Response and Inflammation	CAMP	Cathelicidin antimicrobial peptide has antimicrobial properties and plays a role in the innate immune response by directly killing pathogens and modulating immune cell activity.
	HPT	Haptoglobin binds free hemoglobin released from erythrocytes, thereby preventing oxidative damage and playing a role in the inflammatory response.
		
Vitamin D Metabolism	CYP27B1	This enzyme converts 25-hydroxyvitamin D to its active form, 1,25-dihydroxyvitamin D, which is crucial for calcium homeostasis and immune function.
	CYP24A1	This enzyme degrades active vitamin D, regulating its levels in the body and ensuring a proper calcium and phosphate balance.
	VDR	The vitamin D receptor mediates the effects of vitamin D on gene expression, influencing the immune response and cell proliferation and differentiation.

* According to the HUGO Gene Nomenclature Committee [49].

## Data Availability

The original contributions presented in this study are included in the article/Supplementary Material. Further inquiries can be directed to the corresponding author.

## Supplementary Materials

The supporting information can be downloaded at https://www.mdpi.com/article/10.3390/ijms26010211/s1.
