# Supplementary material for: Differential Expression of Proteins Involved in Skin Barrier Maintenance and Vitamin D Metabolism in Atopic Dermatitis: A Cross-Sectional, Exploratory Study
Source: Int J Mol Sci. 2024 Dec 30;26(1):211. doi: 10.3390/ijms26010211 (PMC11719518; doi:10.3390/ijms26010211)
Supplement: Supplementary file 1 [file ijms-26-00211-s001.zip › Supplementary Figure S1_R1.pdf]

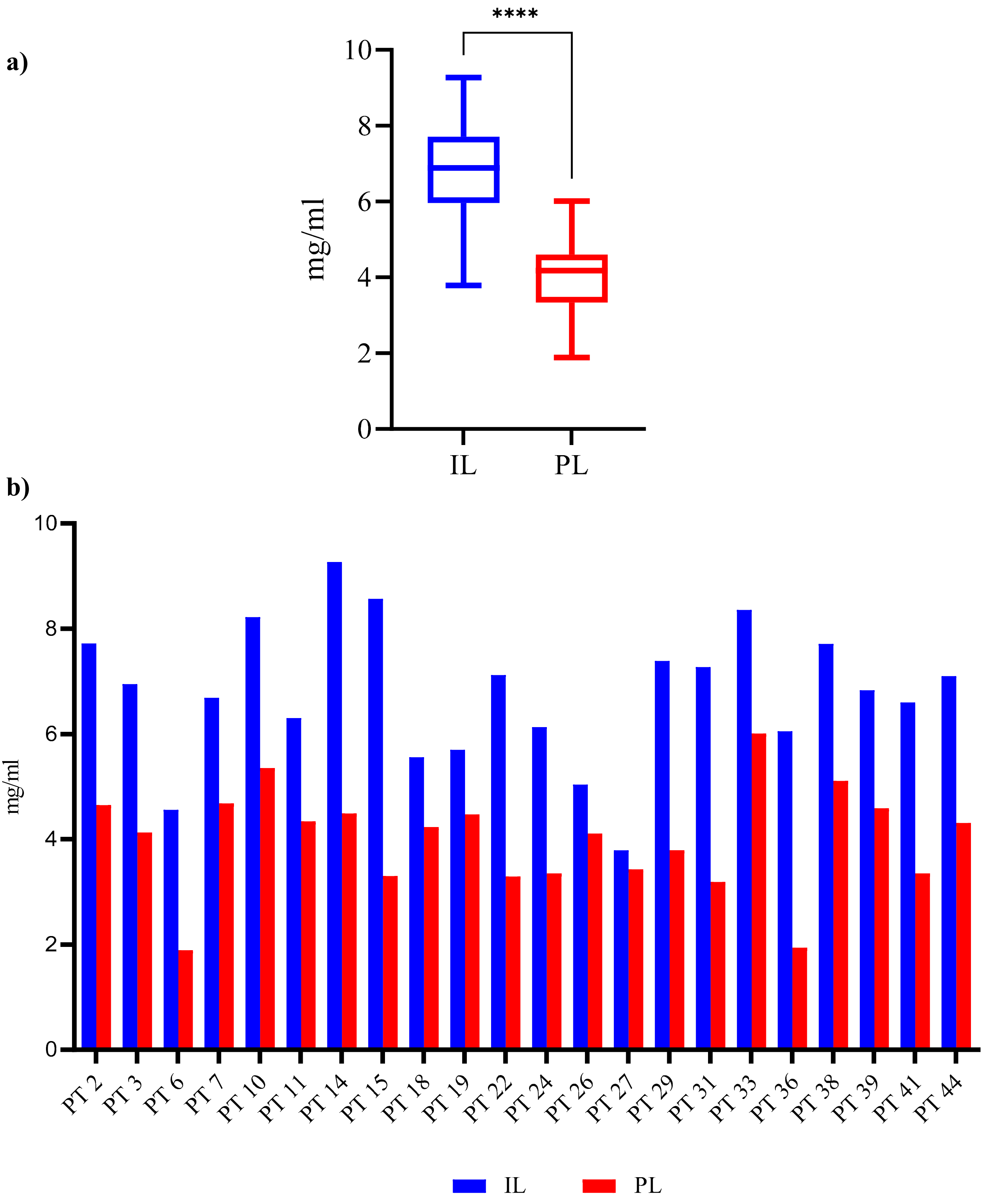

Supplementary Figure S1. Protein concentration distributions in Intra-Lesional (IL) and Peri-Lesional (PL) biopsy groups (a) and for each patient (b).
