# Supplementary material for: Differential Expression of Proteins Involved in Skin Barrier Maintenance and Vitamin D Metabolism in Atopic Dermatitis: A Cross-Sectional, Exploratory Study
Source: Int J Mol Sci. 2024 Dec 30;26(1):211. doi: 10.3390/ijms26010211 (PMC11719518; doi:10.3390/ijms26010211)
Supplement: Supplementary file 1 [file ijms-26-00211-s001.zip › Supplementary Figure S2_R1.pdf]

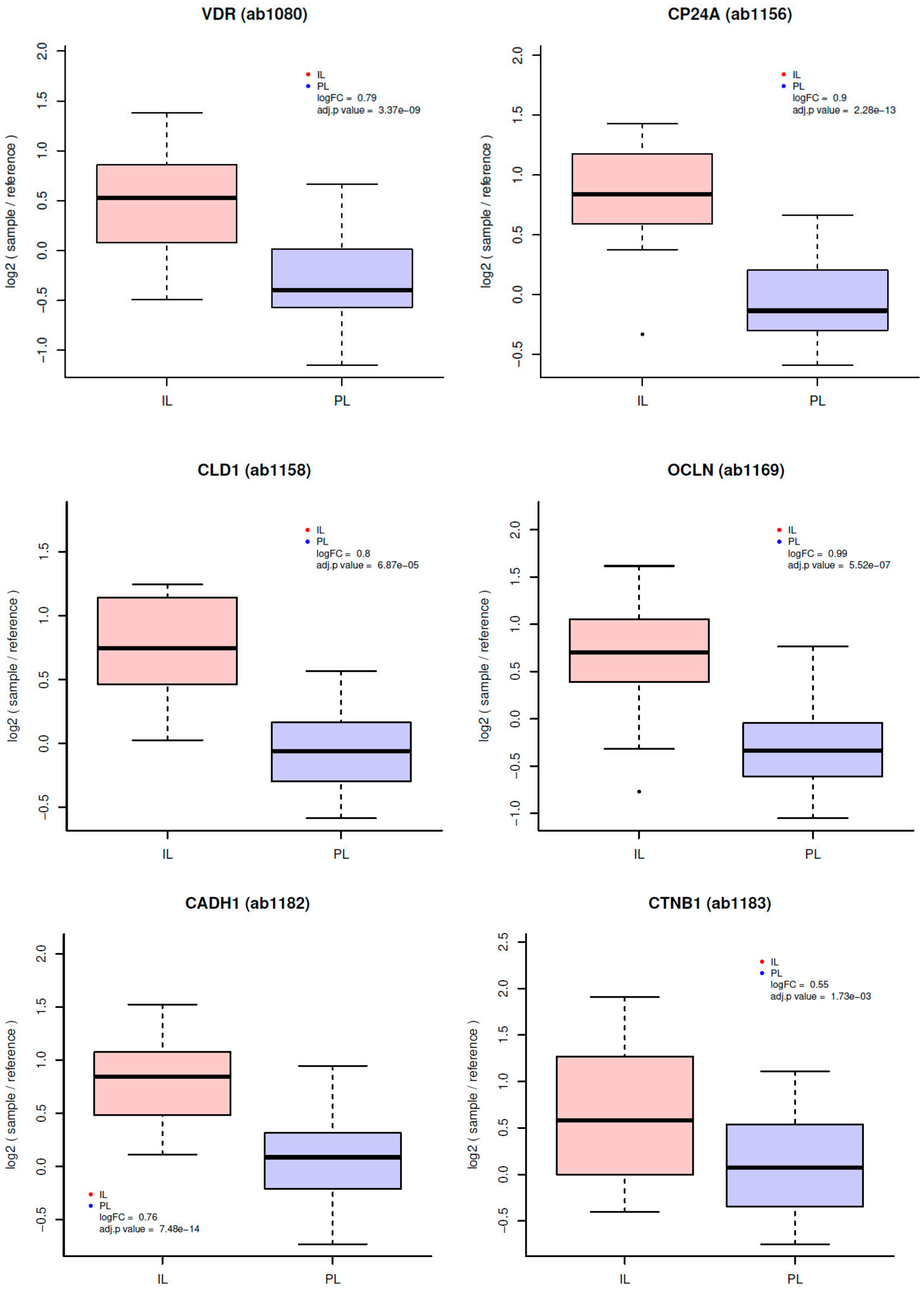
VDR (ab1080)
IL
PL
logFC = 0.79
adj.p value = 3.37e−09
log2 (sample / reference)
IL
PL
CP24A (ab1156)
IL
PL
logFC = 0.9
adj.p value = 2.28e−13
log2 (sample / reference)
IL
PL
CLD1 (ab1158)
IL
PL
logFC = 0.8
adj.p value = 6.87e−05
log2 (sample / reference)
IL
PL
OCLN (ab1169)
IL
PL
logFC = 0.99
adj.p value = 5.52e−07
log2 (sample / reference)
IL
PL
CADH1 (ab1182)
IL
PL
logFC = 0.76
adj.p value = 7.48e−14
log2 (sample / reference)
IL
PL
CTNB1 (ab1183)
IL
PL
logFC = 0.55
adj.p value = 1.73e−03
log2 (sample / reference)
IL
PL

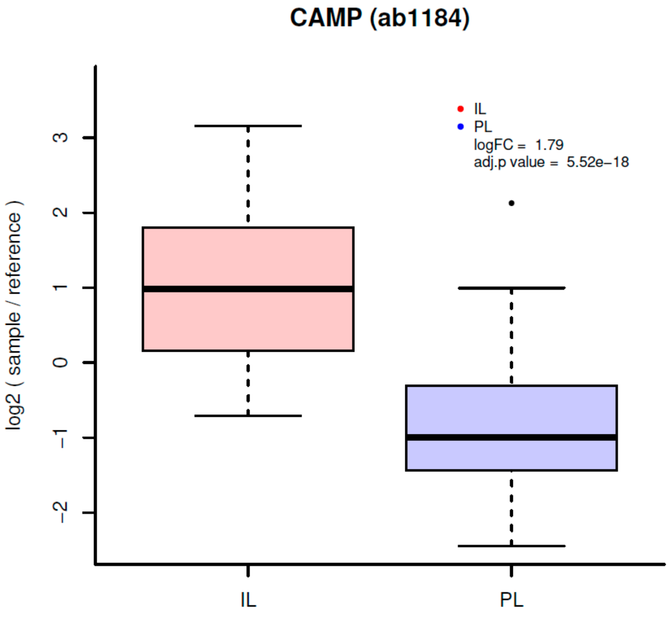
CAMP (ab1184)
log2 (sample / reference)
IL
PL
logFC = 1.79
adj.p value = 5.52e−18

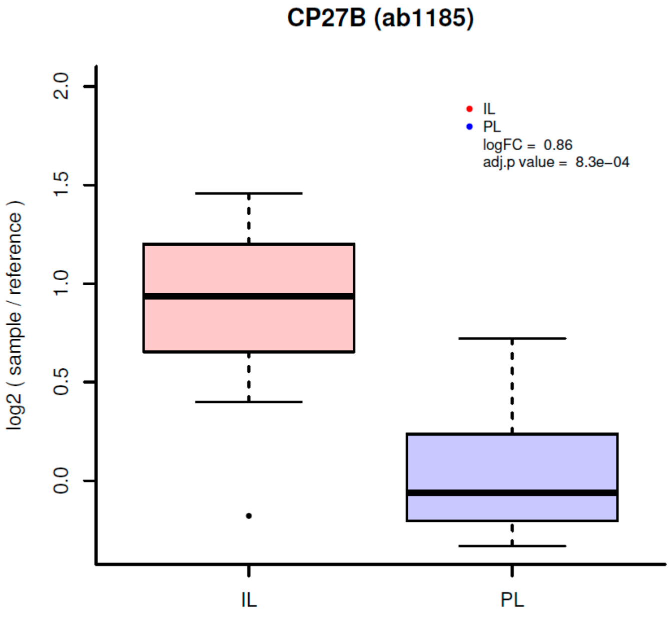
CP27B (ab1185)
log2 (sample / reference)
IL
PL
logFC = 0.86
adj.p value = 8.3e−04

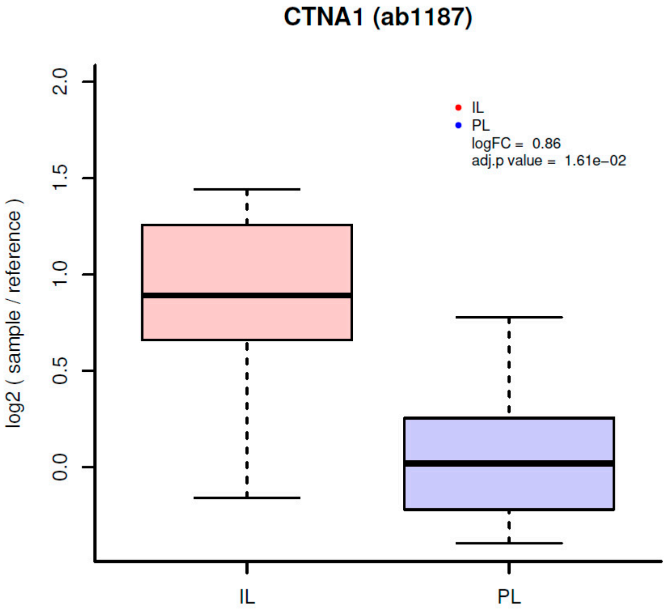
CTNA1 (ab1187)
log2 (sample / reference)
IL
PL
logFC = 0.86
adj.p value = 1.61e−02

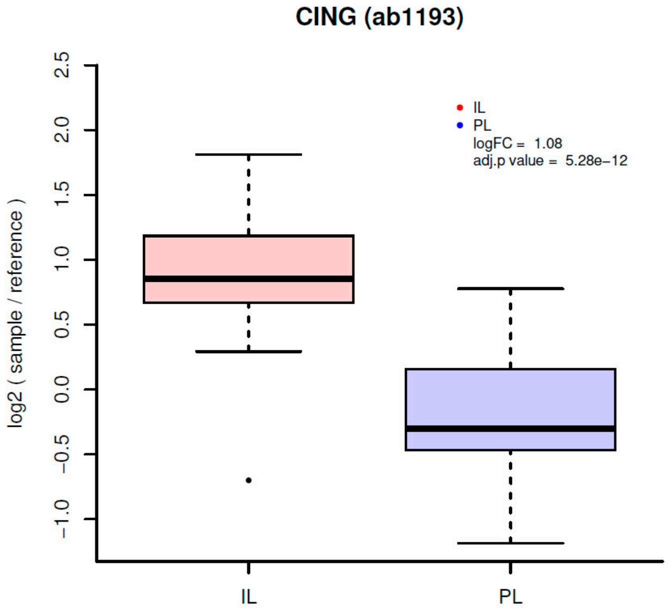
CING (ab1193)
log2 (sample / reference)
IL
PL
logFC = 1.08
adj.p value = 5.28e−12

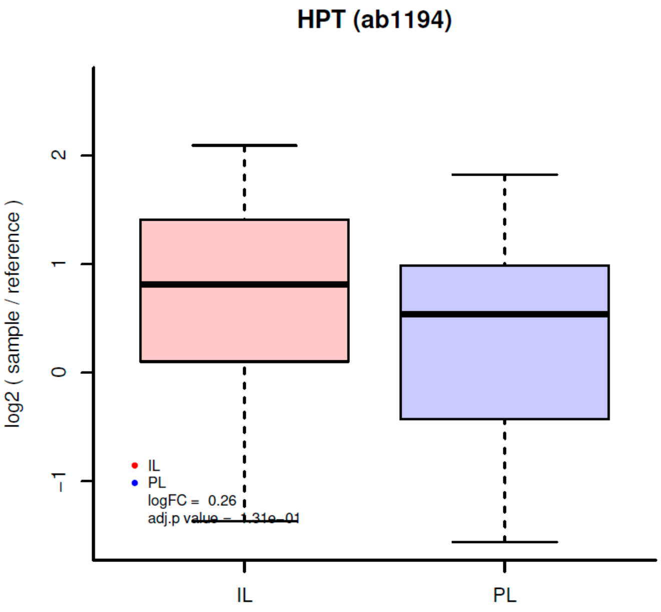
HPT (ab1194)
log2 (sample / reference)
IL
PL
logFC = 0.26
adj.p value = 1.31e−01

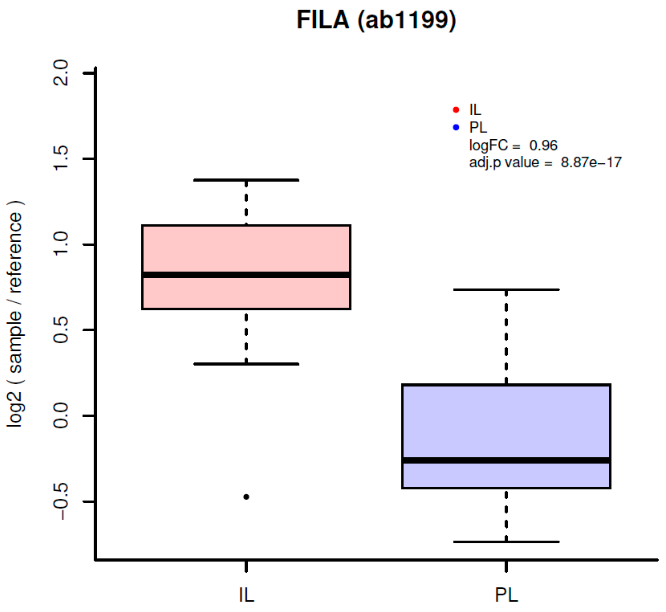
FILA (ab1199)
log2 (sample / reference)
IL
PL
logFC = 0.96
adj.p value = 8.87e−17

Supplementary Figure S2. Box and whisker plots comparing protein expression levels between peri-lesional (PL) and intra-lesional (IL) skin samples in patients with atopic dermatitis. Each box plot represents the distribution of protein expression values for a specific gene. The central horizontal line represents the median and the box represents the interquartile range. The whiskers extend to the most extreme data points that are not considered outliers. CAMP = cathelicidin; CADH1 = Cadherin-1; CING = cingulin; CLD1 = claudin-1; CTNA1 = alpha-catenin; CTNB1 = beta-catenin; CYP24A1 = Cytochrome P450 family 24 subfamily A member 1; CYP27B1 = cytochrome P450 family 27 subfamily B member 1; FILA = filaggrin; HPT = haptoglobin; OCLN = occluding; VDR = vitamin D receptor
