# Supplementary material for: Differential Expression of Proteins Involved in Skin Barrier Maintenance and Vitamin D Metabolism in Atopic Dermatitis: A Cross-Sectional, Exploratory Study
Source: Int J Mol Sci. 2024 Dec 30;26(1):211. doi: 10.3390/ijms26010211 (PMC11719518; doi:10.3390/ijms26010211)
Supplement: Supplementary file 1 [file ijms-26-00211-s001.zip › Supplementary Table S1_R1.pdf]

Supplementary Table S1. Description of skin samples and measured protein concentrations.

Sample ID	Patient ID	Biopsy Group	Protein concentration [mg/ml]	Lesion aspect
DQ001	PT 2	PL	4.65	slight blue
DQ002	PT 2	IL	7.72	slight purple
DQ003	PT 3	PL	4.13	slight purple
DQ004	PT 3	IL	6.95	slight purple
DQ005	PT 6	PL	1.89	clear
DQ006	PT 6	IL	4.56	clear
DQ007	PT 7	PL	4.68	clear
DQ008	PT 7	IL	6.69	slight purple
DQ009	PT 10	PL	5.35	red
DQ010	PT 10	IL	8.22	red
DQ011	PT 11	PL	4.34	slight red
DQ012	PT 11	IL	6.3	slight purple
DQ013	PT 14	PL	4.49	blue
DQ014	PT 14	IL	9.27	blue
DQ015	PT 15	PL	3.3	red
DQ016	PT 15	IL	8.57	red
DQ017	PT 18	PL	4.23	red
DQ018	PT 18	IL	5.56	red
DQ019	PT 19	PL	4.47	red
DQ020	PT 19	IL	5.7	red
DQ021	PT 22	PL	3.29	slight red
DQ022	PT 22	IL	7.12	slight red
DQ023	PT 24	PL	3.35	slight red
DQ024	PT 24	IL	6.13	red
DQ025	PT 26	PL	4.11	red
DQ026	PT 26	IL	5.04	slight red
DQ027	PT 27	PL	3.43	slight red
DQ028	PT 27	IL	3.79	slight red
DQ029	PT 29	PL	3.79	clear
DQ030	PT 29	IL	7.39	red
DQ031	PT 31	PL	3.19	slight red
DQ032	PT 31	IL	7.27	slight red
DQ033	PT 33	PL	6.01	red
DQ034	PT 33	IL	8.36	red
DQ035	PT 36	PL	1.94	clear
DQ036	PT 36	IL	6.05	slight purple
DQ037	PT 38	PL	5.11	slight red
DQ038	PT 38	IL	7.71	brown
DQ039	PT 39	PL	4.59	slight red
DQ040	PT 39	IL	6.83	blue
DQ041	PT 41	PL	3.35	red
DQ042	PT 41	IL	6.6	slight red
DQ043	PT 44	PL	4.31	slight red
DQ044	PT 44	IL	7.1	red

PL, Peri-Lesional sample; IL- Intra-Lesional sample.
