# Supplementary material for: Differential Expression of Proteins Involved in Skin Barrier Maintenance and Vitamin D Metabolism in Atopic Dermatitis: A Cross-Sectional, Exploratory Study
Source: Int J Mol Sci. 2024 Dec 30;26(1):211. doi: 10.3390/ijms26010211 (PMC11719518; doi:10.3390/ijms26010211)
Supplement: Supplementary file 1 [file ijms-26-00211-s001.zip › Supplementary Table S2_R1.pdf]

Supplementary Table S2. Sub-analysis of the correlation between protein expression and clinical features in peri-lesional (PL) areas of patients with atopic dermatitis.

			Vitamin D metabolism			*Epithelial barrier*							*Immune response and inflammation*	
		n	VDR	CPY24A	CPY27B	FILA	CLD1	OCLN	CADH1	CTNNB1	CTNA1	CING	HPT	CAMP
Gender	male	13	-0.3(0.4)	-0.1(0.3)	0.1(0.3)	-0.1(0.3)	-0.1(0.2)	-0.3(0.4)	0.1(0.4)	-0.1(0.5)	0(0.3)	-0.1(0.4)	0.1(0.9)	-0.8(1.2)
	female	9	-0.2(0.5)	-0.1(0.4)	0(0.3)	-0.2(0.4)	-0.1(0.4)	-0.3(0.5)	0(0.5)	0.4(0.5)	0(0.3)	-0.3(0.5)	0.7(1)	-0.8(0.9)
	p-value		*0.31*	*0.34*	*0.20*	*0.24*	*0.40*	*0.50*	*0.23*	***0.032***	*0.50*	*0.21*	*0.095*	*0.48*
Localizzation	not flexural	16	-0.3(0.5)	-0.1(0.3)	0(0.3)	-0.2(0.4)	-0.1(0.3)	-0.3(0.5)	0(0.5)	0.1(0.6)	0(0.3)	-0.2(0.5)	0.4(1)	-0.7(1.2)
	flexural	6	-0.3(0.3)	0(0.2)	0.1(0.3)	-0.1(0.3)	-0.1(0.2)	-0.3(0.3)	0.2(0.3)	0.1(0.5)	0.1(0.3)	-0.1(0.4)	0.2(1)	-1(0.6)
	p-value		*0.49*	*0.24*	*0.21*	*0.28*	*0.48*	*0.46*	*0.10*	*0.46*	*0.39*	*0.29*	*0.32*	*0.33*
25(OH)D levels	≥30 ng/ml	6	-0.4(0.3)	-0.2(0.3)	0(0.3)	-0.2(0.3)	-0.2(0.3)	-0.4(0.3)	0(0.4)	0.1(0.5)	0(0.3)	-0.2(0.3)	-0.1(1.2)	-1(0.9)
	< 30 ng/ml	16	-0.3(0.5)	-0.1(0.3)	0(0.3)	-0.1(0.4)	-0.1(0.3)	-0.3(0.5)	0.1(0.5)	0.1(0.6)	0.1(0.3)	-0.2(0.5)	0.5(0.8)	-0.7(1.1)
	p-value		*0.36*	*0.26*	*0.43*	*0.29*	*0.23*	*0.27*	*0.29*	*0.45*	*0.30*	*0.457*	*0.095*	*0.25*
	≥20 ng/ml	16	-0.2(0.4)	0(0.3)	0.1(0.3)	-0.1(0.4)	0(0.3)	-0.2(0.4)	0.1(0.4)	0.1(0.5)	0.1(0.3)	-0.1(0.4)	0.1(1)	-0.7(1.1)
	< 20 ng/ml	6	-0.4(0.5)	-0.2(0.3)	-0.1(0.2)	-0.4(0.4)	-0.2(0.3)	-0.5(0.5)	-0.2(0.5)	0(0.7)	-0.1(0.2)	-0.4(0.6)	0.9(0.7)	-0.9(1)
	p-value		*0.15*	*0.13*	*0.16*	*0.063*	*0.18*	*0.070*	*0.082*	*0.37*	*0.16*	*0.065*	***0.039***	*0.37*
Skin Prick test	negative	10	-0.4(0.4)	-0.1(0.2)	-0.1(0.2)	-0.2(0.2)	-0.2(0.2)	-0.4(0.3)	0.1(0.4)	-0.1(0.6)	-0.1(0.2)	-0.3(0.3)	0.8(0.7)	-0.5(1)
	positive	12	-0.2(0.5)	0(0.4)	0.1(0.3)	-0.1(0.5)	0(0.4)	-0.2(0.5)	0(0.5)	0.2(0.5)	0.1(0.4)	-0.1(0.5)	0(1)	-1(1.1)
	p-value		*0.16*	*0.23*	*0.087*	*0.27*	*0.083*	*0.26*	*0.46*	*0.10*	*0.058*	*0.24*	***0.015***	*0.13*
IgG levels	≥100 IU/mL	11	-0.3(0.5)	0(0.3)	0.1(0.3)	-0.1(0.4)	0(0.3)	-0.2(0.4)	0(0.5)	0.2(0.6)	0.2(0.3)	-0.1(0.5)	0.1(1)	-1.1(0.9)
	<100 IU/mL	11	-0.3(0.3)	-0.2(0.3)	-0.1(0.2)	-0.2(0.3)	-0.1(0.3)	-0.4(0.4)	0.1(0.3)	0(0.5)	-0.1(0.3)	-0.3(0.4)	0.6(0.9)	-0.5(1.2)
	p-value		*0.36*	*0.12*	*0.071*	*0.15*	*0.17*	*0.14*	*0.24*	*0.19*	***0.035***	*0.18*	*0.14*	*0.074*
EASI	<16	5	-0.1(0.3)	-0.1(0.2)	0(0.2)	-0.2(0.3)	-0.1(0.1)	-0.3(0.3)	0.1(0.3)	0.4(0.6)	0(0.2)	-0.2(0.4)	0.7(1.1)	-0.7(0.5)
	≥16	17	-0.3(0.4)	-0.1(0.3)	0(0.3)	-0.2(0.4)	-0.1(0.3)	-0.3(0.5)	0(0.5)	0(0.5)	0.1(0.3)	-0.2(0.5)	0.3(0.9)	-0.8(1.2)
	p-value		*0.19*	*0.47*	*0.438*	*0.42*	*0.42*	*0.41*	*0.30*	*0.093*	*0.30*	*0.45*	*0.21*	*0.50*

The table reports the expression levels of proteins, involved in vitamin D metabolism, epithelial barrier, immune response and inflammation, stratified by gender, localization, 25(OH)D levels, skin prick test results, IgG levels, EASI score (Eczema Area and Severity Index). p-values < 0.05 are highlighted in bold. Data are presented as mean Log2 (sample/reference) expression and (SD). CAMP = cathelicidin; CADH1 = Cadherin-1; CING = cingulin; CLD1 = claudin-1; CTNA1 = alpha-catenin; CTNB1 = beta-catenin; CYP24A1 = Cytochrome P450 family 24 subfamily A member 1; CYP27B1 = cytochrome P450 family 27 subfamily B member 1; FILA = filaggrin; HPT = haptoglobin; OCLN = occluding; VDR = vitamin D receptor.
