# Supplementary material for: Differential Expression of Proteins Involved in Skin Barrier Maintenance and Vitamin D Metabolism in Atopic Dermatitis: A Cross-Sectional, Exploratory Study
Source: Int J Mol Sci. 2024 Dec 30;26(1):211. doi: 10.3390/ijms26010211 (PMC11719518; doi:10.3390/ijms26010211)
Supplement: Supplementary file 1 [file ijms-26-00211-s001.zip › Supplementary Table S3_R1.pdf]

Supplementary Table S3. Multivariate regression analysis to assess the relationship between protein expression levels in peri-lesional areas and clinical parameters.

		Epithelial barrier							Immune response and inflammation		Vitamin D metabolism		
		OCLN	CING	CLDN1	FILA	CADH1	CTNA1	CTNB1	CAMP	HPT	CYP24A	CYP27B	VDR
Age (< 60 years)	*β* (*95% CI*)	0.74 (-0.26,1.74)	-1.62 (-2.66,-0.58)	0.02 (-0.84,0.88)	n.d.	-0.08 (-0.70,0.54)	0.54 (-1.64,2.72)	-0.32 (-1.02,0.37)	-0.17 (-0.34,0.01)	-0.01 (-0.27,0.25)	1.57 (-0.49,3.63)	-1.25 (-3.66,1.15)	0.35 (-0.75,1.44)
	S.E.	0.45	0.47	0.39	n.d.	0.28	0.98	0.31	0.08	0.12	0.93	1.08	0.49
	T	1.66	-3.46	0.05	n.d.	-0.28	0.55	-1.04	-2.09	-0.1	1.7	-1.16	0.7
	p-value	*0.13*	***0.0061***	*0.96*	n.d.	*0.79*	*0.59*	*0.32*	*0.063*	*0.93*	*0.12*	*0.27*	*0.49*
EASI score (≥16)	*β* (*95% CI*)	-0.20 (-2.10,1.70)	-2.22 (-6.15,1.71)	-0.03 (-1.74,1.68)	4.84 (-4.70,14.38)	-0.22 (-1.28,0.85)	3.99 (0.60,7.37)	-0.52 (-1.78,0.74)	-0.08 (-0.36,0.19)	-0.06 (-0.45,0.34)	-4.22 (-10.33,1.89)	-2.22 (-5.94,1.50)	0.61 (-1.14,2.36)
	S.E.	0.84	1.74	0.76	4.22	0.47	1.50	0.56	0.12	0.18	2.70	1.74	0.77
	T	-0.24	-1.28	-0.04	1.15	-0.46	2.67	-0.93	-0.68	-0.32	-1.56	-1.35	0.79
	p-value	*0.82*	*0.23*	*0.97*	*0.28*	*0.65*	***0.026***	*0.38*	*0.51*	*0.76*	*0.15*	*0.21*	*0.45*
Gender (male)	*β* (*95% CI*)	0.52 (-1.27,2.31)	2.97 (-0.73,6.68)	1.76 (0.15,3.38)	-7.77 (-16.77,1.23)	0.40 (-0.61,1.40)	-3.83 (-7.02,-0.64)	-0.89 (-2.08,0.30)	0.01 (-0.25,0.27)	-0.16 (-0.53,0.22)	4.52 (-1.24,10.29)	2.99 (-0.51,6.50)	-0.25 (-1.90,1.40)
	S.E.	0.79	1.64	0.71	3.98	0.44	1.41	0.53	0.11	0.17	2.55	1.55	0.73
	t	0.66	1.81	2.47	-1.95	0.89	-2.71	-1.69	0.06	-0.96	1.77	1.93	-0.34
	p-value	*0.53*	*0.10*	***0.036***	*0.082*	*0.39*	***0.024***	*0.13*	*0.96*	*0.36*	*0.11*	*0.086*	*0.74*

The table reports only the clinical parameters in which at least one significant association ($p < 0.05$) was observed among the analyzed proteins. Coefficients (β), 95% confidence intervals (CI), and p-values are shown for each variable. n.d., not determined; S.E., standard error: T = T statistic. CAMP = cathelicidin; CADH1 = Cadherin-1; CING = cingulin; CLD1 = claudin-1; CTNA1 = alpha-catenin; CTNB1 = beta-catenin; CYP24A1 = Cytochrome P450 family 24 subfamily A member 1; CYP27B1 = cytochrome P450 family 27 subfamily B member 1; FILA = filaggrin; HPT = haptoglobin; OCLN = occluding; VDR = vitamin D receptor.
